# Actinomykosis

**Published:** 1883-07

**Authors:** J. O’Leary


### THE JOURNAL

OF

### COMPARATIVE MEDICINE AND SURGERY.

VOL. IV. JULY, 1883. No. 3.

### ORIGINAL COMMUNICATIONS.

### ART. XVIII.—ACTINOMYKOSIS.

*Condensed from the writings of Doctor George Fleming in the "Veterinary Journal," and Prof. J. Wortley Axe in the "Veterinarian."*

BY J. O'LEARY, M.D.

THE progress of pathological research is continually demonstrating the mighty part played by microscopic vegetable organisms in the production of disease in plants and animals, generally leading to their destruction, more or less rapidly. The feeblest and smallest as well as the largest and most powerful are alike exposed to the ravages of these invading, relentless foes, whose attack is all the more destructive because it can seldom be detected at its outset.

The immense destruction caused by minute parasitic fungi upon cereal and other useful plants, is only too often experienced by agriculturists and others. The mildew of the wheat, for instance, arises from the attack of a small fungus, "Puccinia graminis." The disease called "smut," attacking the flower of the wheat, is the produce of a minute parasite, "Uredo segetum;" while "bunt," involving the seed itself, is caused by another parasite, "Uredo fœtida." "Ergot," produced by "Spermadia Clavus," attacks rye: and many other destroying fungi, whose existence in plants can only be realized by their ravages, and their presence by means of a microscope, are known to those who have made them a special study.

The lowest as well as the highest animals are similarly the victims of these almost impalpable organisms. The wonderful revelations made by the microscope lead us to believe that those diseases which are included in the group designated "Zymotic" owe their production to vegetable germs; and other maladies not comprised in this class have already been discovered to be due to these microphytes—Anthrax, Tuberculosis, Rabies, etc.

*History.*—As far as the pathology of Actinomykosis is concerned it is a new disease. For a long period it had been observed that the ox tribe was affected with a certain disease of the bones and soft tissues of the head popularly called in Germany "Ladendruck," "Wurm," etc., and in Italy "Mal del rospo" and "Trutta," or Tuberculosis of Tongue. Among veterinarians the disease was looked upon as "Osteosarcoma," "Tuberculosis," "Sarcomatosis," "Lymphoma," etc.

In 1877 Bollinger drew attention to a disease of cattle which he asserted was not infrequent among them, and which consisted of a kind of new formation tumor, that appeared on the upper or lower jaw, in the alveoli of the molar teeth, or sprang from the spongy tissue of the bones, displaced the teeth, and in growing invaded and destroyed the healthy tissues. After some time the round, conglomerate luxuriant growths generally became puriform or ichorous and ulcerated, producing abscesses and fistula, and sometimes increased to the size of a child's head. The progress of the disease was gradual, and interfered with mastication when it advanced to a certain stage; this led to emaciation, and to prevent loss from this cause the owners of the cattle generally had them destroyed before this stage was reached. In examining fresh tumors, Bollinger discovered (in three cases) amongst the dense connective tissue conglomerate masses or nodules of various sizes from that of a walnut to a hen's egg, of soft consistence, pale yellow color, and moist appearance, which on section showed a turbid, whitish-yellow puriform contents; or the nodules were of a spongy texture, in the fine stroma of which were numerous spaces about the dimensions of a hemp-seed, containing a dull-yellow, thick or cheesy-like substance. In scraping a section of an old or young nodule, this substance was easily removed. Microscopically, the tumors appeared to be composed for the greater part of old or embryo granulation tissue, which had a kind of sarcomatous structure, while the cheesy substance consisted of pus corpuscles, granulation and granular cells, as well as fatty granular detritus; in addition, the latter contained innumerable various sized bodies, which were opaque, of a faint yellow tint, often somewhat mulberry-shaped in outline, and here and there encrusted with lime salts. This was recognized as a real fungus, but at first no pathological importance was attached to its presence, and the disease was simply named "Jaw-osteosarcoma."

Besides this noteworthy form, which appeared to have its origin in the invasion of the alveoli by the fungus, the tongue of the ox presented another form proper to itself. Imbedded in the parenchyma of the organ, Bollinger found a number of nodular looking bodies, the majority of which were as large as a millet or hemp-seed, and some as big as a cherry or walnut; many stood prominently from the surface of the mucus membrane. When fresh they were mostly white or grayish-white, diaphonous, moist-looking, very soon becoming turbid or undergoing puriform softening, and vacating their connective tissue capsule. When these nodules were on the upper surface of the tongue, destruction of the mucus membrane, erosion, ulceration, and cicatrization took place: while in the parenchyma of the tongue, a secondary interstitial glossitis became developed, when there was partial atrophy of the muscular fasciculi, and a marked enlargement and wood-like induration of the organ.

The disease, when in the jaws, was not uncommon in old cattle, developing itself in a few weeks, and was nearly always incurable; the animals would survive for a month, or even a year, until the difficulty of eating, because of the diseased jaw or enlarged tongue, produced emaciation and debility, and the animal was slaughtered. In the nodules of the tongue, as in the jaw, the microscopic fungus was constantly present. That the tongue disease was not rare was evidenced by the fact that in one year Bollinger had no fewer than six specimens sent to him from various parts of Bavaria; while in five preparations he had in spirit, he found the fungus. He not only discovered this fungus in the centre of the nodules, but also in the submaxillary lymphatic glands of the tongue, as well as in the tracheal lymphatic glands. He found these glands greatly enlarged and studded with grey and dull-yellowish spongy nodules, in the interior of which he found immense numbers of the fungus. The fungus was likewise discovered in a series of new formation tumors, which cows are very liable to, in the pharynx and larynx, as well as in the mucous membrane of the stomach. In the two former situations, these tumors appeared as polypi and received the name of "Throat-tumor," "Lymphoma," etc.

In fixing upon this endophyte as the cause of the disease, through its destructive nature, and its tendency to produce new formation growths, Bollinger makes some remarks on the fungus, which had been carefully studied by the Professor of Botany at the Munich Veterinary School, Dr. Harz, who obtained it from fresh specimens. The fungus found in the tumors from cattle form globular drussy tufts, from 0, 11 to 1 millimetre in diameter. The majority of these tufts are aggregated in mulberry-shaped masses of from 0, 5 to 1 millimetre in diameter, and appear to the unaided eye as very minute dull-white granules. Very frequently the tufts are somewhat calcareous, and then it is difficult to make out their composition; it is the same when they have become altered by lying some time in alcohol. By a slight pressure made upon it, the fungus tuft is considerably altered in appearance, and mostly assumes the shape of a spheroidal segment, wherein some of the organisms can be distinctly traced throughout. The latter commence at the pointed end of the mass, with a somewhat cone-shaped base-cell, which may possibly represent the non-apparent mycelium, and which bears a large number of short-stalked hyphens. The end of the hyphen shows the Gonidia, which are, like the hyphen itself, polymorphous, and of an oval, globular or elongated form. From the expanded end of the Gonidia are developed a number of young shoots or sprigs, and from each Gonidium arises an individual, so that a number of Gonidia together gives rise to a mulberry-shaped colony; and this is the usual form in which the clusters of fungi appear, though sometimes here and there are found stunted or abortive groups.

The fungus, in fact, is allied in many respects to the common green mould which grows on jam, paste, etc., and is, therefore, very far from being one of the lowest of the group to which it belongs. The individual plant, in reality, consists of a conical mass of branched filaments springing from a single cell, and bearing on their short terminal branchlets the spores or Gonidia by which the mould is produced. From the radiating structure of this micro-entophyte, and its being found at first in the ox tribe, it was named "Actinomyces Bovis." This, Bollinger asserted, was the first instance in which a fungus belonging to the class of moulds had been found in the interior of animal tissues, such as bones.

Since 1868, several Italian and German Veterinarians record observations of much interest and furnish conclusive evidence of the correctness of Bollinger's descriptions and conclusions. I will now briefly sketch the symptoms of the disease, and mention the different situations and animals in which it has hitherto been observed.

*Actinomykosis of the Tongue.*—When the disease is present in the tongue, it is supposed to be Schirrus, Glossitis, Cancer, etc. Doubtless all these morbid states may exist without the presence of the Actinomyces, but I think I may be pardoned, from the cases reported, if I ascribe the majority of the instances which occur of disease in the tongue to this microphyte. In the description given by Captain Russell, F.R.C.V.S., Actinomykosis of the tongue is graphically delineated.

He writes, when treating of induration of the tongue in the ox: "I have observed that the disease commences with small patches of a yellow color, associated with thickening of the mucous membrane, sometimes on the dorsal surface, sometimes on the tip, and at others underneath the tongue, or one or other of its sides. This thickening in the course of a short time breaks up into a number of small pimple-like excrescences, which soon become confluent. As the disease spreads, a cheesy deposit is thrown off, leaving a very red and angry-looking surface. Subsequently, the organ becomes hard and swollen, and eventually hangs from the mouth perfectly useless. The animal quickly loses the power of prehention and deglutition, and if not destroyed usually succumbs to inanition. I do not find that either local or constitutional treatment is of any avail. Four years ago my attention was called to several cases, and in this season I have seen as many as twenty. The progress of the malady is generally slow, the increase in size of the tongue being gradual; but as it progresses movement of the organ is diminished and mastication is performed with corresponding difficulty. There are rarely any indications of severe inflammation noted, and this fact should differentiate the disease from Glossitis, as should also the absence of the acute pain which marks the latter. Discoloration may be present here and there; indeed, this usually precedes ulceration."

These are the chief symptoms when the tongue is the seat of the disease. The pimple-like excrescences, often only the size of a millet-seed, are frequently larger than a walnut. The inflammation and interstitial induration appear to proceed from the surface toward the centre, and the growth of the nodules takes place rapidly; and this is evidently proven from the circumstance attending their reappearance when they have been removed by operation.

In the majority of cases, there are perceived a more or less considerable number of prominences, on the dorsum most frequently, on one or both sides of the tongue, or over the whole of its surface; these look like nodules or tubercules, sometimes like warty excrescences flattened on the top, and vary in size from a millet seed to a walnut, being either single or in clusters. The tongue is enlarged, indurated, "lumpy," often more or less, extensively ulcerated in one or more places; there is very considerable hyperthophy of the submucous and interstitial connective tissue; atrophy or degeneration, more or less marked, of the muscular tissue, and the peculiar yellowish-white round nodules disseminated singly, or in masses throughout, each containing a cluster or at least a tuft of the" Actinomyces." The gums, cheeks, palate, or jaws may also be involved.

An important feature in the micro-pathology of Actinomykosis of the tongue, is the cell infiltration of the connective tissue, and the proliferous multiplication of its nuclear elements and of the muscle nuclei. The new cell forms are as a rule unequally distributed among the muscle fibres, being in some parts widely scattered, in others collected together in dense, rounded, or irregular groups. There is also observable in connection with the latter condition more or less fibrinous exudation, which together with the cell growth have the effect of separating the fibres from each other, and inducing serious textural alterations. New connective tissue, studded with spindle cells, is abundantly present in the structure of the muscle, and by its maturation and contraction leads to atrophy and destruction of its fibres over large areas. By the presence of the inflammatory exudate and embryonic new growth the muscle fibres are squeezed, and may be seen on the borders of the fungus nests flattened out and reduced in their diameter to one-fourth or one-eighth their natural size. Over very considerable areas they are entirely absent, the collapsed sarcolemma only being here or there observable to mark their former presence, while bordering on such parts they seem reduced in size to the dimensions of a blood-corpuscle.

The presence of fungus nests enclosing groups of the actino myces constitutes the most constant histo-pathological feature of the disorder. These peculiar formations may occur in very large numbers, and be closely packed together, or they may be few and far between. I have seen them in every part of the structure of the tongue, but it is in the submucous membrane tissue that they are most frequently and most abundantly found. The nests consists of large masses of cells heaped around smaller or larger colonies of fungi, which they everywhere enclose. They are rounded in form and vary in size according to the strength of the colony and the amount of inflammatory growth present. The cells of which they are composed vary in character under different circumstances. Sometimes they are distinctly epithelioid and contain a simple nucleus, which stains deeply with hamatoxylin, the cell substance remaining clear and faintly distinguishable. In form they are polygonal or rounded. Beyond these which make up the chief bulk of the cell mass, there is a narrow zone of irregular concentric layers of fibro-plastic corpuscles. No intercellular substance intervenes, but under certain conditions, presently to be referred to, branching fragments of degenerated connective tissue are present ramifying through the growth. As each cell mass enlarges it condenses the connective substance around it, and in this way becomes enclosed in a capsule, through which exudation corpuscles are scattered in greater or less amount, as well as the granular remains of cell disintegration. In other and more common examples of these fungous territories the cell elements exhibit a great variety of shape, size, arrangement and condition. Some are round, others hexagonal, oval, rhomboid, etc. They are huddled together without any order whatever into a confused heap, through which are interspersed large numbers of free nuclei, seemingly derived from the proliferous multiplication of connective tissue elements. It is in these examples, which would appear to be formed under a more energetic inflammatory action, that necrosed portions of connective tissue appear within the cell masses. The cells of the fungus territory differ from those of the epitheliomata, not only in their smaller and more uniform size, but also in the fact that they do not form the characteristic dense "concentric globes" or "bird's nest bodies." Like as in tubercle, the cell masses of actinomykosis rapidly undergo degeneration. The change is first seen in the centre of the growths, when the outline of the cells disappear, and they become cloudy and granular, after which they shrink and finally break down into an opaque granular mass. The degenerative process extends from the centre toward the circumference, until the whole is resolved into a caseous condition. The nature of the change involved is essentially fatty degeneration. During the process of cutting and manipulating, the caseous and loose cell masses fall out, when holes with irregular edges are left; similar but more irregular spaces result from the displacement of islets of connective tissue, which have perished in consequence of the blood supply having been cut off.

*Actinomykosis of the Bones of the Jaw.*—Not infrequently we have the tongue and jaws affected simultaneously or consecutively—generally the latter. The tumor which forms on or in the bone is apparently of a sarcomatous or fibro-sarcomatous character, according as the Actinomykosis is periosteal or myeloid. It often commences in the alveoli of the jaw and thence extends into the mouth and the cancellated tissue of the bone, and is accompanied by abscesses and fistula. In this situation it has been observed in the ox, pig, goat and dog.

Only one instance has been recorded in the dog—that by Professor Vachetta, of the Veterinary School at Pisa, and which was published this year under the heading of "Macrocellular Osteo-condrosarcoma, with actinomycosis." About two months before the Professor saw the dog, a swelling appeared, without any assignable cause, on the posterior half of the right branch of the lower jaw, and rapidly increased in volume. In about twenty days the skin became ulcerated, mastication was difficult, and the animal was then brought to the clinic of the school. The ulceration of the skin was now somewhat extensive, and in the centre of this was a small hole, into which a probe could be introduced two or three millimetres. The tumor was hard as a stone at the margin of the dental alveoli, but became softer toward the lower border of the jaw. With the exception of the ulceration, the skin was otherwise healthy in the neighborhood. The tumor was not hot, neither did pressure on it cause pain, but difficulty was evidently experienced in moving the jaw. The mouth was kept half open, and a little saliva flowed from it; the tongue was healthy, and nothing amiss was noticed on the left side or roof of the cavity. The fourth and fifth molars of the right side were pushed upwards by the growth of tumor, and were a little separated from the adjoining teeth. The mucous membrane of the mouth was healthy and the gums were not separated from the teeth. There was no swelling in the intermaxillary space, nor toward the neck. The jaw could be moved passively. The disease was diagnosed as Osteo-sarcoma, probably complicated with myeloplaxy.

In view of the rapid growth of the tumor, and the local and general condition of the animal, as well as the improbability of palliative, or pharmaceutical measures being of any avail, resection of the diseased portion of the jaw was made, and though for some time the prospects of recovery were favorable, yet the dog ultimately succumbed.

The major portion of the tumor was hard and fibrous, and had a yellowish-red tint at the inferior part, whitish elsewhere. At the lower curvature the neoplasm became suddenly and regularly lobulated, the connective tissue forming the interlobular spaces being continuous with that composing the envelope of the tumor as a whole. The inferior third of the section showed multitudes of yellow points, irregularly disseminated throughout; there were none in the upper part. The tumor and its fibrous envelope were very slightly vascular. When examined microscopically, the most important feature noticed was the presence of numerous disseminated Actinomycetes masses, especially toward the inferior part; they were only casually met with in the upper portion, while deeper in the tumor they were very definite in outline, and enclosed in a kind of nucleus composed of apparently dead tissue. Many of these radiate fungi did not show the slightest trace of calcification, others were completely invaded by lime salts, and the nodules enclosing them had to be treated by nitric acid before their contour could be well defined. The fungus appeared in two rather different forms, or rather aspects, which probably depended upon its stage of growth. Cut in the direction of the sarcomatous tissue, intermediate to the necrobiotic focus, there were observed very numerous small discs composed of fine radiating filaments, one portion of which terminated in a rather dark punctiform dilatation. These were more abundant in the peripheral tissue of the tumor, which appeared to contain the younger specimens, and of which there were only a few varieties. There was a more adult form, very often two discs together, in which the radiating filaments, starting from the central discs, were not so slender as in the other example, were of various lengths, and the punctiform dilatations at the end were also larger and more numerous. These dilatations, which may be considered Gonidia in process of maturation, were found in some preparations so developed as to look like true spores, and by their number and minute size they might readily become the active agents of dissemination, far and near, of the micromycetes in the tissues. The other form of Actinomyces was composed of a central irregular, or round disc, light-yellow or olive-tinted and granular, from which proceeded rays much larger than in the preceeding forms. In some of the specimens these rays were approximately equal in length, and altogether the Actinomyces did not look unlike the flower Marguerite. In other instances, the length of the single filaments varied remarkably; while some of these projected only a short distance from the central disc, others extended in a direct and flexous manner right into the surrounding necrobiotic elements. When by pressure the Actinomyces could be separated from each other into single filaments, and these were highly magnified; they were found to be flexible rods, each terminating in a lance-like bulging, or in an angular, single, bifurcated, or trifurcated extremity with a rounded apice. When yet more highly magnified, there was seen in the centre of each filament a fine axial line, either entire, broken, or in points or dots.

Vachetta terminates his observations by remarking that though the canine species has hitherto shown itself refractory to experimental inocculation, yet this instance proves that it may suffer from the accidental disease; that the fungus may present slight variation in form, not only in the different species of creatures in which it has been found (man, ox, pig, horse and dog), but also in individuals and in the different neoplasmata, as is shown by the representations given of it under these circumstances. He was doubtful as to the channel by which it found its way into the tissues—whether by an excoriation, ulceration, or fissure in the gums, or rather by an ulcer of fistulous opening at the lower margin of the jaw.

Johne describes thirteen cases of Actinomykosis of the jaws. Some had a central origin—" Myeloid Actinomykosis," others commenced in the periosteal tissue—" Periosteal Actinomykosis." He also mentions a case of Fibro-Sarcoma of the lower jaw of an ox in which the tumor was the size of two fists, round, fungus and fibrous, and which arose from the alveolar periosteum of the middle incisors; it lay beneath the mucous membrane, and produced great thickening of the lip; another instance of fibrous tumor of the gum, apparently of new formation, the size of a hen's egg, which grew from the periosteum at the interior aspect of the junction of the two portions of the lower jaw, at the lower half of the alveolar border. The stroma of the tumor was three millimetres thick, and the mass, like that of the last tumor, contained "nests" of Actinomycetes. He likewise alludes to an apparently fibro-sarcomatous tumor on the margin of the gum of the lower jaw of a pig: a tumor involving the tongue, about the size of a pigeon's egg, and springing directly from the periosteum on the upper surface of both branches of the jaw. In the more dense fibrous tissue, less in the spongy stroma, were many conglomerations of nodules the size of a millet-seed, containing the Actinomycetes in clusters, many of which were calcified.

Actinomykosis, as it affects osseous tissue, is, I believe, rarely seen in any other bones than those of the face. The great proneness of the maxilla is most likely referable to their intimate relations with the dental apparatus, and more immediately to the laceration and exposure of the alveolar membrane during the shedding of the temporary teeth. The turbinated bones are especially liable by reason of the frequent infection of the Schneiderian membrane from which the disorder extends. When several bones of the face are involved at the same time one or more of the neighboring cavities frequently become encroached upon, and the head presents a large and misshapen appearance. This deformity is due to the mechanical expansion of the osseous texture arising out of an exuberant growth of granulation tissue within it, and the consequent softening, thinning and solution of the ossific lamina. The fungating growth may extend into the sinuses of the head, into the mouth or nasal chambers, either by a process of simple atrophic destruction and perforation of bone, or by an intrinsic carious process, or it may likewise reach the various cavities by interosseous growth. This is especially the case in young animals in whom the sutures are still loose and the bones easily displaced. I have seen the palatine sutures of the superior maxilla, for example, opened out to the extent of an inch by the upward growth of granulation tissue from the palatine membrane, in which case the vomer was completely buried in the new formation and the septum nasi displaced laterally. Loosening and dislodgement of the teeth are common results of actinomykosis of the maxilla. The latter is usually followed by a filling up of the alveolar cavities with soft, vascular, new growths, or portions of the jaw may be exfoliated with the teeth still in the alveoli. The skin and subtegumentary tissues over the region corresponding with the diseased bone are often much indurated, presenting one or more fungating or fistulous wounds, the latter discharging a fœtid sanguinolent pus. When examined after maceration the affected bones present a variety of appearance. In some parts they are but slightly rarefied, in others greatly swollen and distinctly porous, while in more advanced cases they are resolved into a coarse network of delicate plates and fibres, in the midst of which portions of bone are loosely suspended. In this condition the ossific structure will break down under the slightest pressure. In parts removed from the destructive foci of inflammation the constructive effects of exalted nutritive action are seen in the form of tracts of hyperostosis, or thickened and condensed areas of bone.

In certain cases new osseous tissue in the form of one or other of the various osteophytes is developed in the vicinity of the diseased area.

Gamgee (Dairy Stock) undoubtedly alludes to this affection, though he was unaware of its pathology. He writes: "In young cattle there is a somewhat frequent disease termed by some Veterinary Surgeons 'Osteo-Sarcoma,' 'Spina-Ventosa,' and other inappropriate names. The only term I can give it is fibro plastic degeneration of bone. There is no recognized cause of the disease. It occurs most readily from two to five or six years of age, and affects steers in preference to bulls; the lower jaw is most frequently seized in the vicinity of the second or third molar teeth. Sometimes the upper jaw is implicated. . . . . At a spot on the side of the face corresponding to the roots of the third or fourth grinder, above or below, a small, hot, circumscribed swelling occurs. The animal experiences no inconvenience from it, except when the part is struck or pressed upon. The tumor, however, grows and pain increases. In some cases the growth is rapid, and in a few months the disease has invaded the larger part of one-half of the upper or lower jaw, and gives rise to severe symptoms, which arise chiefly from disturbed mastication, pain, and often from various cruel methods of treating the disease, The teeth become loose in their sockets, may be affected by caries, and drop out. Anacker says that sometimes a fistula opens into the mouth. . . . . It is evidently a morbid condition of bony structure. On dissecting the skin off the tumor, we find it covered with tough fibrous tissue arranged in layers. The fibrous element diminishes toward the deeper parts of the growth, where at various parts *yellow accumulations of a friable cellular or granular matter are enclosed in solid cavities*, surrounded by bony plates or a tough gristly tissue. M. Collignon, veterinary inspector of the slaughter house of Montmartre, has observed the disease three times in 300 oxen, and those he found affected came from the marshy plains of La Rochelle. In the plains of Ferrara, and in the Maremme of Tuscany, the disease is very frequent. Low bred animals are most subject to it, and its origin is usually attributed to a blow."

*Actinomykosis of the Fauces and Larynx.*—The disease generally appears in this region in the form of submucous new formations or polypi, which have been classed with the Lymphomata or Lympho-sarcomata. They are round fungus or spongy tumors, covered by apparently normal mucous membrane. There are sometimes several in this situation. They present the same features, histologically, as the nodules in the tongue. Hitherto they have only been found in the ox. Johne describes one of these polypi obtained from the fauces of an ox, as about the size of a fist, round, fungus and soft, covered by normal mucous membrane, rising from the right side of the cavity, a short distance behind the tonsil. On section, it showed five isolated, round and generally fine spongy nodules, the size of a walnut. All of these contained conglomerated masses of the fungus.

The symptoms are difficult in deglutition, and even in respiration, with cough when the tumor is near the laryngeal opening.

Similar tumors are found in the region of the epiglottis and larynx.

*Actinomykosis of the Œsophagus, Lungs, etc.*—A most interesting instance of the disease in the œsophagus is described by Siedamgrotzky, who obtained the specimen fresh and carefully examined it. The mucous membrane of the tube was covered with hundreds of small, flattened, sub-epithelial nodules, from one to four millimetres in diameter, mostly collected in groups, in each of which, in a bright light, a small yellow centre could be distinguished by the naked eye. In some places the small tumors had become confluent to form irregular, compact masses, about twenty millimetres long, of a pale red tint, and in which the yellowish red centres or kernels were visible. Some of the tumors stood out from the mucous membrane like pin's heads. In the middle of the œsophagus was a similarly shaped polypus, eight millimetres in diameter, four millimetres high and four millimetres in diameter at its base on the mucous membrane. The tissue of these masses was yellowish red, soft and filled with numbers of nodules containing the *Actinomyces.*

Two instances of Actinomykosis of the stomach and intestinal canal and two of the udder are described by Johne. Pfluz, of Giesen, describes a case in which the lungs were the seat of the disease, strongly resembling in symptons Pleuro-pneumonia. On microscopic examination the lung substance was found to contain an immense number of tubercules having *Actinomyces* tufts in the centre.

*Pathology.*—There is no doubt as to the eteology of Actinomykosis. The *Actinomyces* is constantly found in new formations of a special kind and through its irritating and disintegrating influence it not only produces these formations, but sets up destructive processes in the tissue in which it may locate itself; and sooner or later, unless it loses its power or is removed from them, causes their death. An *Actinomyces* tumor must, therefore, be looked upon as an "infection tumor" and Actinomykosis as an infectious disease. Mr. Kaufmann, of Lyons Veterinary School, has published some investigations he has made with regard to the infectiveness of the fungus *Aspergillus glaucus.* He proves that the spores of certain moles are as pernicious to the health of animals and mankind as bacterides.

These tumors offer certain distinctive characters, and all tumors containing these characters contain the fungus. Externally, these growths present various appearances, but they are generally round, lobular or fungiform in shape, smooth on the surface and soft in consistency, like the polypi-sarcomata, or somewhat hard like the fibro-sarcomata. In color they also vary, the latter being of a greyish-white, or light yellow tint; the former are darker, less vascular and often stained by blood extravasations. Studding the surface are seen a number of small yellow nodules whose presence is a diagnostic feature. On section the typical character of the Actinomykoma is best displayed. Imbedded in the fibrous stroma are noticed nodules, small and isolated, or in confluent rounded masses the size of a walnut, grey or yellow in color, of a cheesy softness, having minute yellow particles or centres, the clusters of Actinomyces.

The fungus invades the tissues probably by entering, in the form of spores, through a round, abrasion, fissure, or even by means of the delicate mucous follicles of the membrane lining the digestive or respiratory canal.

The species of animal invaded by the *Actinomyces* appears to have much influence on the pathological results. In man, in whom sixteen cases have been recorded, the tendency is to suppurative processes and metastatic abscesses; while in animals it is to new formation tumors and induration or degeneration of tissues.

That the disease is transmissable from one animal to another has been demonstrated experimentally by Johne and Ponfick. There is no record of any instances which might tend to show that the disease may be accidentally transmitted; though the fact that it is inoculable leads us to suppose so. Now that the attention is likely to be directed to the disease by veterinarians and surgeons we may be able to note its accidental transmission from diseased to healthy animals and to mankind.

*Prognosis.*—The prognosis depends on the locality and extent to which it has developed itself. When it is accessible and has not caused serious alteration, and when it can be removed or palliated within a certain time, then it is favorable. Sometimes spontaneous recovery takes place.

*Treatment.*—The treatment of Actinomykosis belongs exclusively to the domain of surgery, and its object must be the extirpation or destruction of the microphyte. This is only possible when it is accessible to the hand, surgical instruments, or destructive agents—caustics, etc. Tumors of the jaws or face can be removed, but it must be for the Veterinary Surgeon to determine as to whether the operation will be profitable, from a pecuniary point of view. Resection of the jaws, a desirable operation in man, is not to be recommended in the case of animals for obvious utilitarian reasons. In all cases operated on care should be taken to dress the wound with such agents as will destroy the spores of the fungus which may chance to remain. When near the surface in the tongue the disease can be destroyed by Carbolic Acid (1 to 25 of water) or Tinct. Fer. Perchlor, Liq. Fer. Perchlor, fort, diluted with only two parts of water.

The sanitary importance of this disease is evident, and it is well to bear in mind that spores of this fungus, alike destructive to man and beast, may invade the body by a trifling scratch or wound, and there set up such changes as to ultimately cause death. Many such cases may have entered our hospitals and come under the observations of the surgeons, without their true nature being suspected; for the disease widely prevails among our cattle, and, therefore, those who come in contact with such creatures must be exposed to accidental transplantation of the *Actinomyces.*

